# Correlation of GABA^+^ levels in the medial prefrontal cortex and circulating follicular helper T cells in neuromyelitis optica spectrum disorder patients with cognitive impairment

**DOI:** 10.1002/brb3.3433

**Published:** 2024-02-21

**Authors:** Yinghui Duan, Qianyun Rui, Yang Yang, Jingluan Tian, Shugang Cao, Feng Zhu, Xiaoyu Duan, Hanqing Gao, Xiaopei Ji, Xinyi Xiao, Yonggang Li, Qun Xue

**Affiliations:** ^1^ Department of Neurology The First Affiliated Hospital of Soochow University Suzhou China; ^2^ Department of Radiology The First Affiliated Hospital of Soochow University Suzhou China; ^3^ Department of Neurology Second People's Hospital of Hefei Hefei Hospital Affiliated to Anhui Medical University Hefei China; ^4^ Institute of Medical Imaging Soochow University Suzhou China; ^5^ Clinical Research Center of Neurology, Jiangsu Institute of Clinical Immunology The First Affiliated Hospital of Soochow University Suzhou China

**Keywords:** cognitive impairment, follicular helper T cells, GABA, neuromyelitis optica

## Abstract

**Background:**

Neuromyelitis optica spectrum disorder (NMOSD) associated with cognitive impairment (CI) is acknowledged. However, the underlying pathogenesis and involvement of the immune system remain unclear.

**Objectives:**

This study aimed to investigate the alterations in immune cells, cytokines, and GABA^+^ levels in NMOSD patients with cognitive deficits.

**Methods:**

Thirty‐eight NMOSD patients and 38 healthy controls (HCs) were included. NMOSD patients were stratified as NMOSD‐CI and NMOSD‐CP groups. The difference in cognitive functions, Tfh and cytokines, and GABA^+^ levels were assessed, and their correlations were calculated.

**Results:**

NMOSD‐CI patients showed worse performance on all cognitive tests, and the percentage of circulating follicular helper T cells (cTfh) was significantly elevated. The frequency of cTfh was positively and negatively correlated with Stroop‐A and AVLT long‐delayed scores, respectively. IL‐21 was remarkably higher in NMOSD‐CI and NMOSD‐CP. The level of GABA^+^ in medial prefrontal cortex (mPFC) was significantly decreased in NMOSD‐CI and was proved positively and negatively correlated with Symbol Digit Modalities Test and the frequency of circulating Tfh cells, respectively.

**Conclusion:**

In NMOSD‐CI patients, all cognitive domains were impacted, , while GABA^+^ levels in mPFC were decreased. GABA^+^ levels in NMOSD‐CI were negatively correlated with the frequency of cTfh, suggesting the underlying coupling mechanism between immune responses and neurotransmitter metabolism in CI in NMOSD patients.

## Graphical abstract

In NMOSD‐CI patients, all cognitive domains were impacted. The frequency of cTfh and IL‐21 was increased, while GABA^+^ levels in mPFC were decreased. GABA^±^ levels in NMOSD‐CI were negatively correlated with the frequency of cTfh.

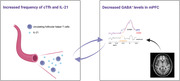

## 1 INTRODUCTION

Neuromyelitis optica spectrum disorder (NMOSD) is an autoimmune inflammatory neurological disease, and the optic nerves and spinal cord are most commonly involved. With the discovery of anti‐aquaporin‐4 antibodies (AQP4‐IgG), NMOSD is considered as an entity independent of multiple sclerosis (MS).

Although NMOSD complicated with cognitive dysfunction is acknowledged, it is still rarely aware of and studied. The prevalence of cognitive impairment (CI) in NMOSD is high heterogeneity, which ranged from 3% to 75% and the consolidated prevalence reached 44% (Moghadasi et al., 2021). To date, no specific and unified tests were identified for the cognitive assessment of NMOSD, this is also one of the issues that causes the high variation of CI in NMOSD. According to a few existing studies, attention, memory, processing speed, verbal fluency, verbal learning, and executive function domains are mostly affected in NMOSD patients (Czarnecka et al., 2020).

About 80% of NMOSD patients were sero‐AQP4‐IgG‐positive (Jarius et al., 2014), indicating a B cell‐mediated humoral immunity. Follicular helper T (Tfh) cells are a subset of CD4^+^ T cells that specially facilitate the formation of germinal center and the development of antibody and memory B cells (Crotty, 2014). The percentage of circulating Tfh cells was found significantly elevated in NMOSD by several studies (Fan et al., 2016; Li et al., 2015; Monteiro et al., 2019; Wu et al., 2021; Yang et al., 2021). However, whether Tfh cells will affect CI in NMOSD patients and the correlation between them still remain unknown.

IL‐21 is a cytokine mainly produced by NKT cells and CD4^+^ T cells and exerts its function by binding to its receptor, IL‐21R. IL‐21 plays an important role in the differentiation of Tfh cells and B cells. Fan et al. (2015) found that plasma IL‐21 level was significantly increased in NMOSD patients. IL‐6 participates in the early stage of Tfh differentiation and the absence of IL‐6 led to early defect of murine Tfh cell differentiation (Crotty, 2014). AQP4‐positive astrocytes were found to produce IL‐6 after exposing to AQP4‐IgG, which was proved to disrupt the function of endothelia cells resulting in the dysfunction of the blood−brain barrier (BBB) (Takeshita et al., 2017). High levels of IL‐6 (Feng et al., 2023) and IL‐21 (Agrawal et al., 2022) were also proved to be associated with cognitive decline, but their roles in the CI of NMOSD are unclear.

The optimal cognitive function relies on the balance of excitatory/inhibitory (E/I) network. Studies on cognitive deficits were mostly concentrated on the excitatory (glutamatergic and cholinergic) pathways; however, the dysfunction of the inhibitory (GABAergic) signaling pathway of E/I balance also contributed to the pathogenesis of Alzheimer's disease (AD) (Bi et al., 2020; Calvo‐Flores Guzmán et al., 2018). The level of GABA can be measured in vivo using magnetic resonance spectroscopy (MRS), a powerful noninvasive technique for brain metabolites measurements. The decrease of GABA level was confirmed in AD and Alzheimer's disease (MCI) patients by several studies (Jiménez‐Balado & Eich, 2021). Our team first performed the study to investigate the changes and correlations of neurotransmitters and cognitive functions in NMOSD patients, and we found that GABA level was significantly decreased in the medial prefrontal cortex (mPFC) in NMOSD patients and was positively correlated with overall cognition and verbal memory (Yang et al., 2022). However, in this study, all NMOSD patients were mixed together without stratification into CI and cognitive preserved (CP) groups.

Therefore, in this study, we aim to investigate the relationship between cognitive deficits and immune cells, cytokines, and GABA levels with NMOSD patients stratified according to their cognitive performances.

## 2 METHODS

### 2.1 Participants

Thirty‐eight patients with NMOSD were consecutively recruited from the Department of Neurology of First Affiliated Hospital of Soochow University between September 2020 and December 2022. The inclusion criteria were patients (1) met the Wingerchuk 2015 diagnostic criteria for NMOSD; (2) between 18 and 70 years; (3) right‐handedness; (4) relapse‐free in the last 4 weeks; and (5) provided written informed consent. Thirty‐eight volunteers, whose age, gender, handedness, and education were matched to the NMOSD patients, were recruited from the local community as healthy controls (HCs). The exclusion criteria for both NMOSD patients and HCs were: (1) taking high‐dose steroids or antianxiety drugs; (2) contraindications to MRI scanning; (3) a history of other concomitant disorders that affect cognition; (4) severe visual and hearing impairments that could affect the cognitive function tests; and (5) diagnosed with severe depression or anxiety (HAMA ≥ 21; HAMD ≥ 24). The severity of anxiety and depressive symptoms of all participants was evaluated with the Hamilton Anxiety rating scale (HAMA) and Hamilton Depression rating scale (HAMD), respectively. This study was approved by the Ethics Committee of Soochow University and written informed consents were obtained from all participants.

### 2.2 Cognitive functions

Within 1 week of the MRI scanning, the cognitive functions of all participants were evaluated by a well‐trained experienced neurologist. The Mini‐Mental State Examination (MMSE) and the Montreal Cognitive Assessment (MoCA) were used to detect global CI. Specific cognitive domains were also assessed with the following tests: (1) Symbol Digit Modalities Test (SDMT) for cognitive processing speed measurement; (2) Stroop Color and Word tests (SCWT) and Trail‐making test part B (TMT‐B) for executive function analysis; (3) Trail‐making test part A (TMT‐A) for attention evaluation; (4) Clock Drawing Test (CDT) for visual‐spatial function measurement; (5) Controlled Oral Word Association Test (COWAT) for verbal fluency evaluation; and (6) Auditory‐Verbal Learning Test‐Huashan version (AVLT‐H) for verbal memory evaluation, which included immediate recall, short‐term delayed recall, long‐term delayed recall, cued recall, and recognition.

Raw scores of all cognitive function tests were transformed into z‐scores. NMOSD patients were considered as CI when z‐scores were below or above 1.5 standard deviations of HCs in two or more two cognitive domains. The other NMOSD patients were defined as CP.

### 2.3 Immunological information

#### 2.3.1 Antibodies and flowcytometry

Peripheral blood (sodium‐heparinized) was obtained from all participants on the day of MRI scanning and stored at 4°C, flowcytometry was conducted within 6 h. Corresponding antibodies were added to 50 μL blood samples and incubated at 4°C for 30 min. Two hundred microliters Erythrolytic reagent (Beckman Coulter) were then added and incubated in a 37°C water‐bath for 10 min. The sample was washed with 1 mL PBS and centrifuged for 5 min at 1500 rpm. The supernatant was discarded and 500 μL PBS was used to suspend the cell pellet. The cell suspension was analyzed on a Navios EX (Beckman Coulter) and flow cytometric data analyses were done with FlowJo 10. For flowcytometry analysis, the following antibodies were utilized: anti‐CD3 (clone OKT3, BioLegend), anti‐CD4‐PC7 (clone SFCI12T4D11, Beckman Coulter), and anti‐CXCR5‐PE (clone J252D4, BioLegend).

#### 2.3.2 Enzyme‐linked immunosorbent assay

Plasm was collected by centrifuging peripheral blood (sodium‐heparinized) for 3 min at 1000 rpm. The levels of IL‐6 and IL‐21 were measured with ELISA kits (Sigma) according to the manufacturer's protocols. The level and titration of AQP4‐IgG were measured with ElisaRSR AQP4 Ab Version 2 kit (RSR Ltd).

### 2.4 MRS acquisition

**FIGURE 1:**
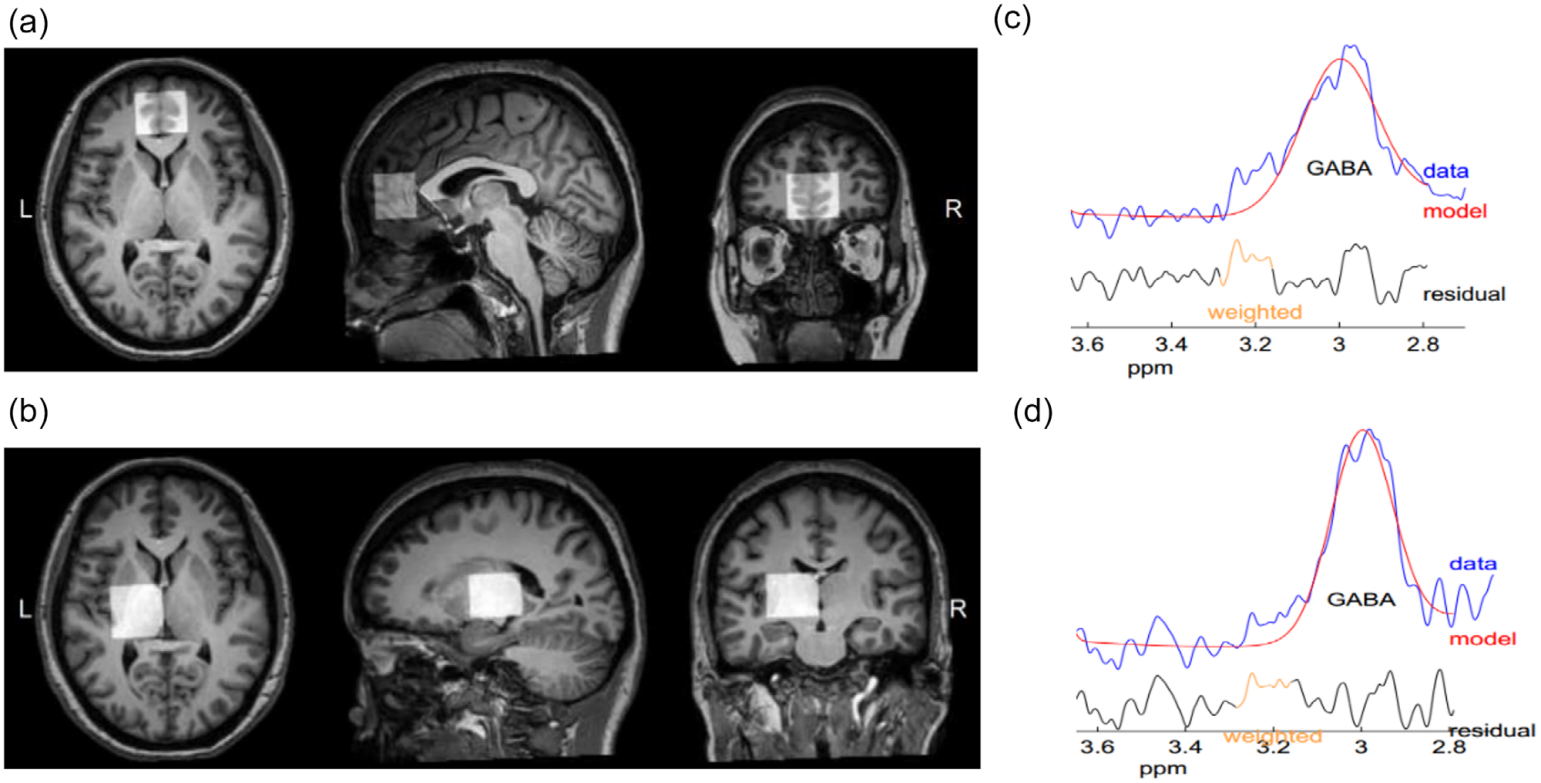
Location of VOIs and sample spectral fitting. (A) and (B) show the location and size of VOIs in the mPFC and left thalamus in axial, sagittal, and coronal positions, respectively. The GABA peak resonate at 3.02 ppm, (C) and (D) display the spectral fitting in the mPFC and left thalamus, respectively, with residual in black, sample data in blue, and fit model in red.

All participants underwent MRI examinations using a 3.0T MRI scanner (Philips Ingenia, Philips Healthcare). The brain three‐dimensional high‐resolution T1‐weighted images were collected, and the detailed parameters used for data collection were as follows: (a) repetition time = 7.0 ms; (b) echo time = 3.1 ms; (c) slice thickness = 1 mm; (d) field of view = 256 × 256 × 185 mm^3^; (e) flip angle = 8°; (f) voxel size = 1 × 1 × 1 mm^3^. mPFC and thalamus were thought to be critical areas for cognitive processing and were applied in previous studies. Therefore, these two areas were chosen in this study and the exact locations were positioned as described in our previous study (Figure 1A,B) (Yang et al., 2022). MEGA‐PRESS sequence was used to acquire the level of GABA and total creatine (tCr). Parameters of MEGA‐PRESS were as follows: (a) repetition time = 2000 ms; (b) echo time = 68 ms; (c) 320 averages; (d) data points = 2048; (e) spectral width = 2 kHz; (f) variable power and optimized relaxations delays (VAPOR) water suppression. During odd and even number acquisitions, editing pulses (14 ms) were applied at 1.89 and 7.46 ppm, which were defined as ON experiment and OFF experiment, respectively. The GABA signal was obtained at 3.02 ppm by subtracting the spectra from ON experiment from OFF experiment (Mullins et al., 2014). The tCr signal was obtained at 3.0 ppm from the spectra of OFF experiment. Since the signal at 3.02 ppm contained not only GABA, but also some homocarnosine and macromolecule, this signal was referred to as GABA^+^ in this study.

To postprocess the MRS data, the Gannet 3.0 toolkit was used with default parameters. The Gaussian model and Lorentzian model were used to fit GABA^+^ and tCr signals, respectively. The MRS data were output as the ratio of GABA^+^ to tCr (GABA^+^/tCr). Individual metabolic peak was excluded if the signal fitting error was above 15%.

### 2.5 Statistical analysis

SPSS software (version 26.0) was used for statistical analyses of demographic, clinical, and neuropsychological data. For continuous variables, the Shapiro−Wilk test was performed to verify the normal distribution. The differences in ages, education duration, disease duration, EDSS, number of relapses, neuropsychological tests, and level of IL‐21 between groups were assessed using the Mann−Whitney U‐test. The difference in gender and AQP4‐IgG between groups was analyzed by Fisher's exact test. *t*‐Test was used to evaluate the difference in the frequencies of CD4^+^CXCR5^+^ T cells, level of IL‐6, and GABA^+^/Cr ratio between groups. The Kruskal−Wallis H test was used to analyze the difference in age, education duration, HAMA, and HAMD among three groups. A general linear model was used to assess the difference in neuropsychological tests, immune characteristics, and GABA^+^/Cr among three groups, with age, education duration, and gender as covariates. Bonferroni correction was used for post‐hoc comparisons. For all the correlation analyses in this study, the Spearman correlation coefficient was utilized, with age, gender, and education duration corrected. *p* < .05 was set as the statistical significance level.

## 3 RESULTS

### 3.1 Demographic and clinical characteristics

Thirty‐eight NMOSD patients and 38 HCs were recruited in this study. Among NMOSD patients, 18 (47.5%) were defined as CI patients and 20 (52.5%) as cognitively preserved after assessing their cognitive functional tests. The demographic and clinical data of all participants are summarized in Table 1 and Supplementary 2. The NMOSD‐CI patients had older age (*p* = .005), shorter education duration (*p* = .001), higher HAMA scores (*p *= .003), and higher HAMD scores (*p* < .001) than HC; however, no significant differences are found between NMOSD‐CP and HC. The disease duration, number of relapses, and AQP4‐IgG data showed no difference between NMOSD‐CI and NMOSD‐CP, while higher EDSS scores (*p* = .002), older age (*p *< .001), and lower education (*p* < .001) are observed in the NMOSD‐CI group. No significant difference was identified in gender among the three groups. In the NMOSD‐CI and NMOSD‐CP groups, eight and seven patients were reported hypoplasia, respectively.

**TABLE 1:** Demographic and clinical characteristics of NNOSD‐CI, NMOSD‐CP, and HC.

	NMOSD‐CI (*n* = 18)	NMOSD‐CP (*n* = 20)	HC (*n* = 38)	*p‐value* (three groups)	*p*_1_ (NMOSD‐CI vs. HC)	*p*_2_ (NMOSD‐CP vs. HC)	*p*_3_ (NMOSD‐CI vs. NMOSD‐CP)
Gender (F/M)	12/6	17/3	26/12	.333			
Age (years)	56.50 (45.00, 65.25)	33.00 (27.50, 45.75)	46.50 (26.50, 53.25)	.001	.005	.121	< .001
Education duration (years)	5.50 (1.75, 9.00)	14.00 (10.25, 16.00)	11.50 (7.75, 17.00)	< .001	.001	.748	< .001
Disease duration (months)	27.0 (23.00, 48.50)	36.00 (15.00, 45.00)	NA	.849			
Number of relapses	3.00 (2.00, 4.25)	2.00 (2.00, 3.75)	NA	.347			
EDSS	3.00 (2.50, 4.00)	2.00 (1.00, 2.50)	NA	.002			
HAMA	4.00 (1.75, 5.25)	2.00 (1.00, 4.00)	1.00 (0.00, 4.00)	.010	.003	.115	.132
HAMD	3.50 (1.75, 6.25)	1.50 (0.25, 3.75)	0.50 (0.00, 2.25)	.003	< .001	.090	.096
AQP4‐IgG (positive /negative)	11/7	11/9	NA	.752			

*Note*: Categorical data are presented as numbers, and continuous data as median and IQR.

Abbreviations: AQP4‐IgG, aquaporin‐4 immunoglobulin G; CI, cognitively impaired patients; CP, cognitively preserved patients; EDSS, Expanded Disability Status Scale; HAMA, Hamilton Anxiety Scale; HAMD, Hamilton Depression Scale; HC, healthy controls; NA, not applicable; NMOSD, neuromyelitis optica spectrum disorder.

### 3.2 Cognitive functional characteristics

Table 2 summarizes the neuropsychological characteristics of all participants. Compared to the NMOSD‐CI group, both NMOSD‐CP and HC groups showed better performance on global (MMSE and MoCA) and all specific (SDMT, Stroop, TMT‐B, TMT‐A, CDT‐30, COWAT, and AVLT) cognitive functional tests. However, no significant difference of cognitive tests was identified between NMOSD‐CP and HC groups.

**TABLE 2:** Neuropsychological characteristics of NNOSD‐CI, NMOSD‐CP, and HC.

	NMOSD‐CI (*n* = 18)	NMOSD‐CP (*n* = 20)	HC (*n* = 38)	*p‐value* (three groups)	*p*_1_ (NMOSD‐CI vs. HC)	*p*_2_ (NMOSD‐CP vs. HC)	*p*_3_ (NMOSD‐CI vs. NMOSD‐CP)
MMSE	27.50 (26.75, 28.00)	29.00 (28.00, 30.00)	29.00 (28.00, 30.00)	< .001	< .001	.906	< .001
MoCA	24.50 (23.00, 26.00)	28.00 (27.00, 28.75)	28.00 (26.00, 30.00)	< .001	< .001	.789	< .001
SDMT	21.00 (18.25, 30.25)	47.50 (37.75, 54.00)	48.00 (37.00, 55.50)	< .001	< .001	.900	< .001
Stroop‐A, s	55.57 ± 22.37	38.40 ± 14.01	37.23 ± 5.35	< .001	< .001	.985	< .001
Stroop‐B, s	72.56 ± 23.73	49.72 ± 10.32	43.15 ± 8.05	< .001	< .001	.247	< .001
Stroop‐C, s	121.93 ± 36.37	91.38 ± 26.45	78.74 ± 9.69	< .001	< .001	.149	< .001
TMT‐B, s	150.000 (108.928, 190.928)	60.060 (50.445, 75.215)	65.145 (45.418, 85.838)	< .001	< .001	.656	< .001
TMT‐A, s	23.025 (13.525, 40.215)	9.205 (6.378, 12.208)	8.75 (6.475, 12.335)	< .001	< .001	.875	< .001
CDT‐30	24.00 (22.75, 28.00)	27.00 (24.50, 29.50)	28.00 (26.00, 30.00)	< .001	< .001	.144	.036
COWAT	38.50 (27.00, 46.75)	50.00 (40.25, 57.00)	48.00 (40.00, 59.00)	.001	.002	.887	.003
AVLT immediate	18.50 (15.75, 20.50)	24.00 (18.25, 26.75)	23.50 (19.75, 27.25)	< .001	< .001	.767	.001
AVLT short‐delayed	7.00 (4.00, 7.00)	9.00 (7.25, 9.00)	9.00 (6.75, 10.00)	< .001	.001	.744	< .001
AVLT long‐delayed	5.00 (3.75, 6.00)	8.00 (7.25, 9.00)	8.00 (6.00, 10.00)	< .001	< .001	.423	< .001
AVLT cued recall	4.00 (3.00, 5.25)	7.50 (6.00, 10.00)	7.00 (6.00, 10.00)	< .001	< .001	.666	< .001
AVLT recognition	20.00 (18.00, 21.25)	22.00 (22.00, 23.75)	22.00 (21.00, 23.00)	< .001	.002	.359	< .001

*Note*: Data are presented as mean ± SD or median and IQR.

Abbreviations: AVLT, Auditory Verbal Learning Test; CDT, Clock Drawing Test; CI, cognitively impaired patients; COWAT, Controlled Oral Word Association Test; CP, cognitively preserved patients; HC, healthy controls; MMSE, mini‐mental status examination; MoCA, Montreal cognitive assessment; NMOSD, neuromyelitis optica spectrum disorder; SDMT, Symbol Digit Modalities Test; Stroop, Stroop Color and Word tests; TMT, Trail‐making test.

### 3.3 The involvement of immune cells and cytokines

Peripheral blood was obtained from 16 NMOSD‐CI patients, 19 NMOSD‐CP patients, and 16 HC, and flowcytometry and ELISA were performed subsequently. The percentage of CD4^+^CXCR5^+^T cells was significantly elevated when compared to NMOSD‐CP and HC groups (gating strategy refers to Supplementary 1), and the level of IL‐21 was remarkably higher in both NMOSD‐CI and NMOSD‐CP groups than HC (Table 3). However, the level of IL‐6 showed no significant difference among the groups. The correlation of CD4^+^CXCR5^+^T cells and IL‐21 was analyzed in the NMOSD‐CI group. The frequency of CD4^+^CXCR5^+^ T cells was positively correlated with Stroop‐A scores (*r* = 0.381, *p* = .045) and negatively correlated with AVLT long‐delayed scores (*r* = −0.381, *p *= .045), with age, education duration, and EDSS corrected (Table 4). The level of IL‐21 showed no correlation with neuropsychological tests.

**TABLE 3:** The characteristics of Tfh and cytokines in NMOSD and HC groups.

	NMOSD‐CI (*n* = 16)	NMOSD‐CP (*n* = 19)	HC (*n* = 16)	*p‐value* (three groups)	*p*_1_ (NMOSD‐CI vs. HC)	*p*_2_ (NMOSD‐CP vs. HC)	*p*_3_ (NMOSD‐CI vs. NMOSD‐CP)
CD4^+^CXCR5^+^ T cells (%)	10.44 ± 3.61	7.25 ± 4.04	4.90 ± 2.16	< .001	< .001	.144	.025
IL‐6 (pg/mL)	46.01 ± 9.16	41.40 ± 8.29	45.68 ± 3.65	.133	.228	.296	1.000
IL‐21 (pg/mL)	883.83 (768.18, 908.05)	770.34 (675.06, 849.44)	245.65 (209.58, 343.75)	< .001	.001	.001	.925

*Note*: Data are presented as mean ± SD or median (IQR).

Abbreviations: CI, cognitively impaired patients; CP, cognitively preserved patients; HC, healthy controls; NMOSD, neuromyelitis optica spectrum disorder.

**TABLE 4:** Correlation of Tfh with neuropsychological tests.

	CD4^+^CXCR5^+^ T cells (%)		IL‐21 (pg/mL)	
	*r*	*p*	*r*	*p*
MMSE	−0.247	.205	0.174	.375
MoCA	−0.237	.224	−0.018	.926
SDMT	−0.180	.361	−0.077	.696
Stroop‐A	0.381	.045	0.105	.595
Stroop‐B	0.240	.219	0.143	.469
Stroop‐C	0.205	.296	−0.143	.468
TMT‐B	0.185	.345	0.074	.709
TMT‐A	0.096	.627	−0.024	.904
CDT‐30	−0.021	.915	−0.214	.275
COWAT	−0.335	.082	−0.136	.489
AVLT immediate	−0.045	.819	−0.171	.383
AVLT short‐delayed	−0.274	.158	0.004	.985
AVLT long‐delayed	−0.381	.045	−0.076	.700
AVLT cued recall	−0.330	.086	−0.025	.900
AVLT recognition	−0.358	.061	−0.043	.827

Abbreviations: AVLT, Auditory Verbal Learning Test; CDT, Clock Drawing Test; CI, cognitively impaired patients; COWAT, Controlled Oral Word Association Test; CP, cognitively preserved patients; HC, healthy controls; MMSE, mini‐mental status examination; MoCA, Montreal cognitive assessment; NMOSD, neuromyelitis optica spectrum disorder; SDMT, Symbol Digit Modalities Test; Stroop, Stroop Color and Word tests; TMT, Trail‐making test.

### 3.4 Difference of GABA^+^/Cr between groups and correlation analysis

The mPFC and left thalamus were chosen for data acquisition. The MR spectra images of a representative NMOSD patient are shown in Figure 1A,B. The representative GABA^+^ peaks in the mPFC and left thalamus are displayed in Figure 1C,D.

Due to data quality issues, such as baseline instability, 9 NMOSD patients and 13 HCs were excluded from the mPFC group, and 9 patients and 10 HCs were excluded from the left thalamus group. Eventually, 14 NMOSD‐CI patients, 15 NMOSD‐CP patients, and 25 HCs were included in the mPFC group, where six patients had hypoplasia in the NMOSD‐CI group, meanwhile, 15 NMOSD‐CI patients, 14 NMOSD‐CP patients, and 28 HCs were included in the left thalamus group. As shown in Table 5, the level of GABA^+^/Cr in the mPFC was significantly decreased in NMOSD‐CI patients when compared to HC (*p* = .023), while there is no remarkable difference in the left thalamus between groups.

**TABLE 5:** Difference in GABA^+^/Cr between groups.

	NMOSD‐CI (*n* = 14)	NMOSD‐CP (*n* = 15)	HC (*n* = 25)	*p‐value* (three groups)	*p*_1_ (NMOSD‐CI vs. HC)	*p*_2_ (NMOSD‐CP vs. HC)	*p*_3_ (NMOSD‐CI vs. NMOSD‐CP)
mPFC	0.0914 ± 0.0209	0.1022 ± 0.0396	0.1272 ± 0.0451	.018	.023	.160	1.000
	NMOSD‐CI (*n* = 15)	NMOSD‐CP (*n* = 14)	HC (*n* = 28)	*p‐value* (three groups)	*p*_1_ (NMOSD‐CI vs. HC)	*p*_2_ (NMOSD‐CP vs. HC)	*p*_3_ (NMOSD‐CI vs. NMOSD‐CP)
Left thalamus	0.1276 ± 0.0189	0.1326 ± 0.0201	0.1243 ± 0.1778	.979			

*Note*: Data are presented as mean ± SD.

Abbreviations: CI, cognitively impaired patients; CP, cognitively preserved patients; GABA^+^, GABA plus co‐edited macromolecules; HC, healthy controls; mPFC, medial prefrontal cortex; NMOSD, neuromyelitis optica spectrum disorder.

The correlation analysis of GABA^+^/Cr in the mPFC of the NMOSD‐CI group with neuropsychological and immune characteristics was performed, with age, gender, and education duration corrected. GABA^+^/Cr was proved positively correlated with SDMT scores (*r* = 0.489, *p* = .018) and had a negative correlation with the frequency of CD4^+^CXCR5^+^ T cells (*r* = −0.413, *p* = .026) (Table 6).

**TABLE 6:** Correlation of GABA^+^/Cr with neuropsychological and immune characteristics.

	*r*	*p*		*r*	*p*
Cognitive tests					
MMSE	0.063	.774	CDT‐30	−0.072	.745
MoCA	0.118	.592	COWAT	0.091	.680
SDMT	0.489	.018	AVLT immediate	−0.182	.405
Stroop‐A	−0.319	.137	AVLT short‐delayed	0.308	.153
Stroop‐B	−0.080	.716	AVLT long‐delayed	0.317	.141
Stroop‐C	−0.243	.263	AVLT cued recall	0.322	.134
TMT‐B	0.013	.954	AVLT recognition	0.107	.629
TMT‐A	−0.014	.948			
Immune characteristics					
CD4^+^CXCR5^+^ T cells (%)	−0.413	.026	IL‐21 (pg/mL)	0.107	.581
IL‐6 (pg/mL)	0.202	.294			

Abbreviations: AVLT, Auditory Verbal Learning Test; CDT, Clock Drawing Test; CI, cognitively impaired patients; COWAT, Controlled Oral Word Association Test; CP, cognitively preserved patients; HC, healthy controls; MMSE, mini‐mental status examination; MoCA, Montreal cognitive assessment; NMOSD, neuromyelitis optica spectrum disorder; SDMT, Symbol Digit Modalities Test; Stroop, Stroop Color and Word tests; TMT, Trail‐making test.

## 4 DISCUSSION

Since Blanc first reported the association of NMOSD with CI in 2008 (Blanc et al., 2008), more research was performed in this field. The prevalence of CI in NMOSD ranged from 3% to 75% and the consolidated prevalence reached 44% (Moghadasi et al., 2021). In our study, 47.4% (18/38) of NMOSD patients suffered from cognitive deficits, they were prone to severe disability, depression, and anxiety. However, younger age and longer education duration seem to protect NMOSD patients from CI, which is in line with previous studies (Kong et al., 2022; Yang et al., 2022). Kim and colleagues also confirmed shorter education years in NMOSD‐CI patients, but they found no statistic difference in age and EDSS between groups (Kim et al., 2017).

In our study, NMOSD‐CI patients showed significant impairment in all cognitive domains, that is cognitive processing speed, executive function, attention, visual‐spatial function, verbal fluency, and verbal memory. Although different cognitive tests were used, the impairment of all cognitive domains was also observed in NMOSD‐CI patients by other researchers (Kim et al., 2017).

About 80% of NMOSD patients were sero‐AQP4‐IgG‐positive (Jarius et al., 2014), indicating a B cell‐mediated humoral immunity. Depletion of B cells with rituximab and inebilizumab was frequently used to prevent relapse. Rituximab was found to induce the fluctuation of Tfh and PD‐1^+^CXCR5‐CD4^+^ T peripheral helper (Tph) cells, which can both contribute to plasma cell differentiation and antibody production (Huang et al., 2023). Tfh cells are essential for the differentiation of B cells to antibody‐secreting cells; moreover, Tfh cells were presumed to play an important role in autoantibodies generation and contributed to autoimmune diseases (Tangye et al., 2013). The frequency of circulating CD4^+^CXCR5^+^ T cells was found to increase in systemic lupus erythematosus, Sjögren's syndrome, rheumatoid arthritis, juvenile dermatomyositis, Graves’ disease, and myasthenia gravis (Tangye et al., 2013). Although Li et al. (2019) did not find any difference, more studies confirmed the increase of circulating Tfh cells in NMOSD (Fan et al., 2016; Li et al., 2015; Monteiro et al., 2019; Wu et al., 2021; Yang et al., 2021; Zhao et al., 2017). In our study, CD4^+^CXCR5^+^ T cells were remarkably elevated in NMOSD‐CI but not in the NMOSD‐CP group when compared with HCs. This indicates that Tfh cells may participate in the pathogenesis of cognitive deficits in NMOSD. Moreover, the correlation analysis revealed that the frequency of circulating CD4^+^CXCR5^+^ T cells was positively and negatively correlated with Stroop‐A and AVLT long‐delayed, respectively.

Although many cytokines were involved in Tfh generation, IL‐6 and IL‐21 are the initial ones (Tangye et al., 2013). IL‐6 secreted by dendritic cells promotes Tfh cell differentiation and the generation of AQP4‐IgG is also conducted by an IL‐6‐dependent B cell subpopulation (Chihara et al., 2011). AQP4‐positive astrocytes were found to produce IL‐6 after exposing to AQP4‐IgG, which was proved to disrupt the function of endothelia cells resulting in the dysfunction of BBB (Takeshita et al., 2017). Two humanized monoclonal antibodies (tocilizumab and satralizumab) targeting IL‐6R were proved to be efficacious by clinical trials in sero‐AQP4‐IgG‐positive patients (Tugizova et al., 2021). IL‐6 was found to increase in the serum of NMO patients (Chihara et al., 2011), but no differences were identified between groups in our study. Wei and colleagues found that the level of IL‐6 was significantly elevated in cerebrospinal fluid (CSF) but not in the serum of first‐onset NMOSD patients, especially AQP4‐IgG positive patients (Wei et al., 2018), which indicates that the changes of IL‐6 are confined to the CNS. Plasma level of IL‐6 was also found upregulated, but it is only in relapsing NMOSD patients not in remitting patients (Zhao et al., 2017). However, in our study, only serum IL‐6 was measured, and the patients included were all in a relapse‐free state. This might be the possible interpretation of our results.

Our study first demonstrates the underlying immune mechanism of NMOSD patients associated with CI. The increased percentage of circulating Tfh cells and IL‐21 level in NMOSD‐CI patients indicates that activation of the immune system is associated with CI in NMOSD. Levels of IL‐21, as well as circulating Tfh, were elevated in AD patients and mice (Agrawal et al., 2022). Moreover, this study also showed that microglia activation and Aβ deposition were enhanced via IL‐21 injection and inhibited by blocking IL‐21R in 5xFAD mice, suggesting the important role of IL‐21 in the pathogenesis CI. Plasma level of IL‐21 was found significantly increased in NMOSD patients (Fan et al., 2016; Fan et al., 2015; Li et al., 2015), which is a coincidence with our study. Zhao and colleagues performed deeper research and found that the plasma level of IL‐21 was upregulated in the relapse phase but not in the remission state, and B cell depletion with rituximab (RTX) could reduce the level of IL‐21 (Zhao et al., 2017). However, in our study, all patients were in remission state, and more patients in the NMOSD‐CP group (12/20) received rituximab than the NMOSD‐CI group (4/18), which could lead to a lower IL‐21 level in the NMOSD‐CP group. Therefore, whether the elevated IL‐21 level in the NMOSD‐CI group resulted from the disease pathogenesis or the impact of rituximab administration remains doubtful. The exact mechanism and pathways in NMOSD patients remain unclear and more deep investigations are still needed.

The optimal cognitive function relies on the balance of the E/I network. Our team first performed the study to investigate the changes of neurotransmitters in NMOSD patients with cognitive functions, and we found that GABA level was significantly decreased, while excitatory glutamate and glutamine (Glx) neurotransmitter showed no statistic difference between groups (Yang et al., 2022). In present study, mPFC and thalamus were chosen to measure neurotransmitter levels, which were critical areas in normal cognitive processing (Halassa & Kastner, 2017; Li et al., 2022) and used in previous MS (Cao et al., 2020; Kantorová et al., 2022) and NMOSD (Liu et al., 2018; Wang et al., 2015; Yang et al., 2022) studies. Our previous findings showed that GABA^+^ levels were decreased in mPFC in NMOSD patients, but no difference was observed in the thalamus. By stratifying NMOSD patients to CI and CP groups in the present study, we identified that the GABA^+^ levels were only descended in NMOSD‐CI groups. Resulting in its physiological capacity in up‐taking and converting glutamate to glutamine, which is the substrate for glutamatergic and GABAergic neurons to generate glutamate and GABA, respectively, astrocytes play a crucial and dominant role in maintaining the integration of glutamate/GABA‐glutamine cycle (Andersen et al., 2022). AQP4 expressed on astrocytes’ end‐feet form complex with glutamate transporters (mainly excitatory amino acid transporter 2 [EAAT2]) and are the main targets of AQP4‐IgG in NMOSD patients (Liu et al., 2018). The interaction of AQP4‐IgG and AQP4 on astrocytes leads to the disruption of astrocytes and the downregulation of EAAT2 (Abe & Yasui, 2022; da Silva et al., 2019). This results in the accumulation of glutamate in extracellular space and less GABA generation, leading to an imbalance of the E/I network which is the exacerbating or even pathogenic factor in the pathogenesis of AD (Czapski & Strosznajder, 2021; Pajarillo et al., 2019). This might be an explanation for reduced GABA^+^ levels in NMOSD patients associated with CI. In addition, Tfh cells were found to contribute to the pathophysiology in an NMOSD mouse model, and Tfh cell depletion via abrogating ICOS/ICOS‐L signaling ameliorated astrocytopathy (Yick et al., 2023). Tfh cells were also found positively correlated with severity (Monteiro et al., 2019; Yang et al., 2021) and relapse (Cheng et al., 2022; Fan et al., 2016; Zhao et al., 2017) in NMOSD patients. Fan and colleagues concurrently confirmed the positive correlation of percentages of CCR7‐ and CCR7‐ICOS^+^ memory Tfh cells with AQP4‐IgG level (Fan et al., 2016). Therefore, Tfh cells may also contribute to the reduction of GABA^+^ levels through mediating more generation of AQP4‐IgG to induce astrocytopathy, which is consistent with our finding that GABA^+^/tCr levels in mPFC in NMOSD‐CI were negatively correlated with frequency of CD4^+^CXCR5^+^ T cells. Different from our previous study (Yang et al., 2022) that GABA^+^/tCr levels in mPFC were positively correlated with overall cognition and verbal memory in NMOSD, herein we found it was only positively correlated with information processing speed (SDMT) in NMOSD‐CI. Prefrontal cortical GABAergic interneurons are proved crucial for memory execution by selectively processing different inputs, and mPFC is the convergent target of multiple long‐range inputs and plays the role in filtering essential information from numerous signals (Delli Pizzi et al., 2017; Yang et al., 2021). Although Thielen and colleagues did not find a correlation between GABA levels in mPFC and memory performance (Thielen et al., 2018), the decreased GABA levels in mPFC theoretically impact the information processing process, leading to a worse cognitive execution speed.

Although a German prospective multicenter study performed intraindividual cognitive tests for 217 NMOSD patients during a 2‐year follow‐up and no significant cognitive worsening was observed (Hümmert et al., 2023), CI is dynamic and needs long‐term observation. This study was a single‐center cross‐sectional study, and the sample size was small. Therefore, larger‐scale multicenter long‐term follow‐up studies are needed and more immune cells and cytokines should be measured to better understand the immune mechanism of NMOSD with CI.

Except for the mentioned small sample size, there are some other limitations in our study. First, by stratifying NMOSD patients to CI and CP groups, the size of HC was almost double the size of NMOSD‐CI or NMOSD‐CP groups. Although corrections for multiple comparisons were carried out during statistic, the unbalanced group stratification still may affect the results. Second, optic neuritis is the main symptom in NMOSD patients and affects their visual acuity which will impact their ability to perform cognitive assessment; however, the visual acuity of NMOSD patients was not performed. Besides, fatigue is a common complaint in 58−77% of NMOSD patients (Seok et al., 2022), which would impact their ability in daily activities. In this study, several cognitive tests were performed, but the fatigue severity of NMOSD patients was not assessed. Third, the difference in education duration between NMOSD‐CI and NMOSD‐CP groups was significant, which will affect the results in cognitive and psychological performances. Fourth, although MRS has been increasingly used to measure neurotransmitter levels in vivo, it still has limitations. MRS is capable of precisely detecting the total levels of GABA and Glx in the volume of interest (VOI), but the location and size of VOI varied from studies. Besides, it cannot distinguish between intra‐ and extracellular GABA and Glx levels, moreover, between glutamine and glutamate, which are composed of the Glx (Li et al., 2022; Pasanta et al., 2023). Last but not least, education duration can affect cognitive performance. Although we recruited HC and NMOSD patients who matched in education duration, but after stratifying, the education duration was significantly lower in the NMOSD‐CI group than in the NMOSD‐CP and HC groups, which could lead to worse cognitive performance of the NMOSD‐CI group.

To conclude, our study first investigated the underlying immune mechanism of CI in NMOSD patients by stratifying patients to CI and CP groups. We found that in NMOSD‐CI patients all cognitive domains were impacted, the frequency of CD4^+^CXCR5^+^ T cells and IL‐21 levels was increased, while GABA^+^ levels were decreased in mPFC. Besides, GABA^+^ levels in the NMOSD‐CI group were negatively correlated with the frequency of CD4^+^CXCR5^+^ T cells, suggesting the underlying coupling mechanism between immune responses and neurotransmitter metabolism in cognition impairment in NMOSD patients.

## AUTHOR CONTRIBUTIONS

**Yinghui Duan**: Conceptualization; methodology; writing―original draft; visualization; formal analysis; data curation. **Qianyun Rui**: Methodology; software; data curation; formal analysis. **Yang Yang**: Methodology; software; data curation; visualization; formal analysis. **Jingluan Tian**: Methodology; data curation; formal analysis. **Shugang Cao**: Methodology; data curation; formal analysis. **Feng Zhu**: Software; data curation; methodology. **Xiaoyu Duan**: Data curation; resources; visualization. **Hanqing Gao**: Data curation; resources. **Xiaopei Ji**: Data curation; resources. **Xinyi Xiao**: Data curation; resources. **Yonggang Li**: Conceptualization; methodology; writing―review and editing; software; funding acquisition; supervision. **Qun Xue**: Conceptualization; methodology; writing―review and editing; funding acquisition; supervision.

## CONFLICT OF INTEREST STATEMENT

All authors declare that the research was conducted in the absence of any commercial or financial relationships that could be construed as a potential conflict of interest.

### PEER REVIEW

The peer review history for this article is available at https://publons.com/publon/10.1002/brb3.3433.

## Supporting information

Supplementary 1. The gating strategy of Tfh cells. The peripheral blood was stained with anti‐CD3, anti‐CD4, and anti‐CXCR5 antibodies to identify Tfh cells, which is CD4^+^CXCR5^+^ cell population.
Supplementary 2 Clinical features of NNOSD patients.

## Data Availability

Data not provided in the article because of space limitations may be shared (anonymized) at the request of any qualified investigator for purposes of replicating procedures and results.
