# Supplementary material for: Correlation of GABA+ levels in the medial prefrontal cortex and circulating follicular helper T cells in neuromyelitis optica spectrum disorder patients with cognitive impairment
Source: Brain Behav. 2024 Feb 21;14(2):e3433. doi: 10.1002/brb3.3433 (PMC10881283; doi:10.1002/brb3.3433)
Supplement: Supplementary file 1 — Supplementary 1. The gating strategy of Tfh cells. The peripheral blood was stained with anti‐CD3, anti‐CD4, and anti‐CXCR5 antibodies to identify Tfh cells, which is CD4+CXCR5+ cell population. Supplementary 2 Clinical features of NNOSD patients. [file BRB3-14-e3433-s001.docx]

| 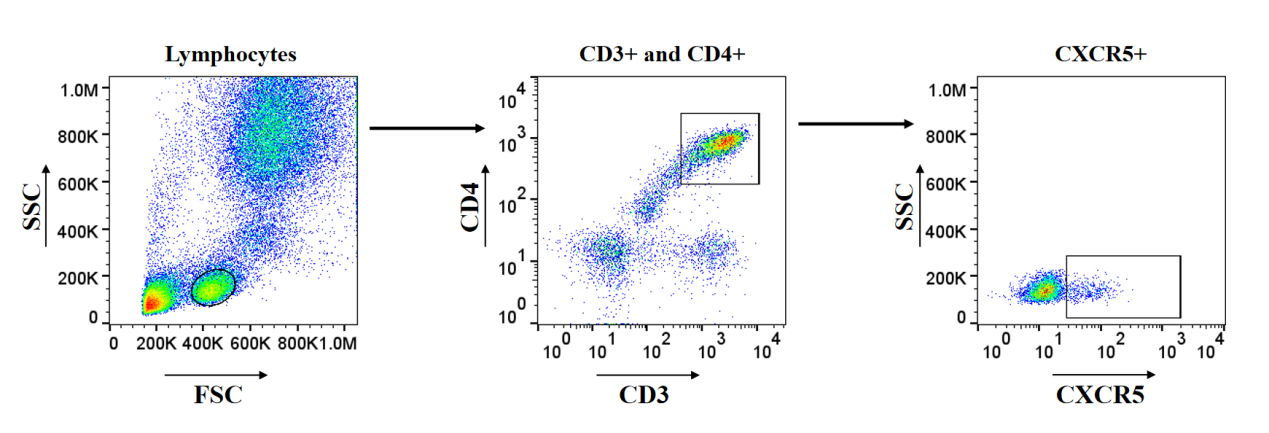 |
| --- |
| **Supplementary 1. The gating strategy of Tfh cells.** The peripheral blood were stained with anti-CD3, anti-CD4, and anti-CXCR5 antibodies to identify Tfh cells, which is CD4+CXCR5+ cell population. |

| **Supplementary 2 Clinical features of NNOSD patients.** | | | | | | |
| --- | --- | --- | --- | --- | --- | --- |
| **Group** | **AQP4-IgG** | **MOG-IgG** | **Core criteria** | **Location of lesions** | **Treatment** | **Relative drugs used** |
| **NMO-CP** | + | - | ON (right eye), Acute myelitis (chest and back pain, bladder and bowel dysfunction) | brain MRI (-), C2 and T1-T4 | prednisone, AZA |  |
|  | + | NA | Acute brainstem syndrome | left middle cerebellar peduncle | prednisone |  |
|  | + | NA | Acute myelitis (Weakness of the limbs), APS | C2-C5, dorsal medulla | prednisone, MMF | Baclofen, Oxcarbazepine |
|  | + | - | ON (both eyes), APS, Acute myelitis | multiple lesions in the cervical and thoracic segments, area postrema lesions | RTX |  |
|  | + | - | Acute myelitis (Weakness of the legs) | T4-7 |  |  |
|  | + | NA | ON (right eye) | brain and spine MRI (-) | prednisone |  |
|  | - | NA | ON (left eye) Acute myelitis (back pain) | brain MRI (-), T3-5 and T6-T9 | RTX |  |
|  | - | - | ON (both eyes) Acute myelitis (Weakness of the legs, numbness below nipple line) | optic nerves, C6-T2 and T4-T6 | prednisone, RTX |  |
|  | + | NA | ON (right eye) | brain and spine MRI (-) | RTX |  |
|  | + | - | APS | area postrema lesions | RTX |  |
|  | + | - | ON (right eye), Acute myelitis(Weakness and numbness of the legs) | C2-C3 and T3-T6, brain MRI (-) | prednisone |  |
|  | - | NA | Acute myelitis(numbness of the legs), APS | T5-8, area postrema lesions | prednisone | Pregabalin |
|  | - | NA | Acute myelitis(Weakness and numbness of the limbs), APS | dorsal medulla, spine MRI (-) | RTX |  |
|  | - | - | ON (left eye), APS | area postrema lesions | RTX |  |
|  | - | - | Acute myelitis(numbness of the limbs) Acute brainstem syndrome | C2-C6, pons | RTX |  |
|  | - | NA | ON (both eyes), Acute myelitis (numbness of the limbs) | brain MRI (-), C3-C7 | RTX |  |
|  | - | - | Acute myelitis (pain of the legs), APS | area postrema lesions, multiple lesions in the cervical and thoracic segments | RTX | Baclofen, Pregabalin |
|  | + | NA | Acute myelitis (Weakness of the limbs) | dorsal medulla, multiple lesions in the cervical segments | RTX |  |
|  | + | - | ON (both eyes), APS, Acute myelitis | area postrema lesions, multiple lesions in the cervical segments | RTX |  |
|  | - | NA | ON (both eyes) Acute myelitis (Weakness and numbness of the limbs) | brain MRI (-), C3-6 | prednisone | Baclofen, Pregabalin |
| **NMO-CI** | - | NA | ON (both eyes) Acute myelitis (Weakness of the limbs) | C3-C6 and T1-T10, brain MRI (-) | AZA | Gabapentin, Oxcarbazepine |
|  | + | NA | Acute myelitis (Weakness of the legs) | T3-6 | AZA | Baclofen, Pregabalin |
|  | + | - | Acute myelitis (Weakness and numbness of the limbs), APS | area postrema lesions, C2-4, C7 and T2-3 | prednisone, AZA | Pregabalin |
|  | + | - | ON (left eye) Acute myelitis (numbness of the limbs) | brain MRI (-), C4-6 | AZA |  |
|  | - | NA | ON (left eye), Acute myelitis (Weakness and numbness of the limbs) | brain MRI (-), T6-8 and T9 | prednisone |  |
|  | - | NA | Acute myelitis (Weakness and numbness of the limbs), APS | C3-C5 and T3-T7, dorsal medulla | CTX | Celecoxib, Olanzapine |
|  | + | - | ON (both eyes) | brain and spine MRI (-) | prednisone |  |
|  | - | + | ON (both eyes), Acute myelitis(Weakness of the legs) | brain MRI (-), T3-7 | prednisone |  |
|  | + | - | Acute myelitis (Weakness of the legs), APS | brain MRI (-), multiple lesions in the cervical segments | RTX | Oxcarbazepine |
|  | + | NA | Acute myelitis(Weakness and numbness of the limbs) | optic nerves, C3-5 | MMF |  |
|  | + | NA | Acute myelitis(Weakness of the legs),APS | area postrema lesions, T8-T12 | prednisone |  |
|  | - | - | Acute myelitis (Weakness and numbness of the limbs), Symptomatic cerebral syndrome with NMOSD-typical brain lesion(s) | dorsal thalamus, multiple lesions in the centra semiovale, C4-6 and T4-7 | CTX |  |
|  | + | NA | ON (right eye) | brain and spine MRI (-) | RTX |  |
|  | + | NA | Acute myelitis (Weakness and numbness of the legs) | T3-6 | RTX | Pregabalin, escitalopram oxalate |
|  | + | - | Acute myelitis (Weakness and numbness of the limbs) | brain MRI (-), T10-12 | RTX | Oxcarbazepine, Baclofen |
|  | + | - | ON (right eye), APS, Acute myelitis | dorsal medulla, right middle cerebellar peduncle，C3-4, T1-3 and T9-10 | Methylprednisolone |  |
|  | - | NA | ON (right eye), APS | dorsal medulla, spine MRI (-) | Methylprednisolone |  |
|  | - | NA | ON (right eye) Acute myelitis (numbness and pain of the legs) | brain MRI (-), C2-5 | CTX | Oxcarbazepine, Baclofen |
| Abbreviations: [ON](https://en.wikipedia.org/wiki/Optic_neuritis): Optic neuritis; CTX: Cyclophosphamide; APS: Area Postrema Syndrome; RTX: Rituximab; AZA: Azathioprine; MMF: mycophenolate mofetil; NA: not available | | | | | | |
